# Preparation and In Vitro Bioactivity Evaluation of *Ganoderma lucidum* Melanin-Stabilized Selenium Nanoparticles

**DOI:** 10.3390/foods15020250

**Published:** 2026-01-09

**Authors:** Ruru Liu, Qunluo Cao, Heng Miao, Yuting Pei, Guoqing Wei, Yanfen Cheng, Xueran Geng, Junlong Meng, Mingchang Chang, Lijing Xu

**Affiliations:** College of Food Science and Engineering, Shanxi Agricultural University, Taigu, Jinzhong 030801, China; 19935424814@163.com (R.L.); 18103488573@163.com (Q.C.); 15035594432@163.com (H.M.); 18035451767@163.com (Y.P.); 15663740812@163.com (G.W.); cyf2341986@163.com (Y.C.); gengxueran2007@163.com (X.G.); mengjunlongseth@126.com (J.M.)

**Keywords:** selenium nanoparticles, *Ganoderma lucidum*, melanin, stability, anti-inflammatory activity, antioxidant activity

## Abstract

Selenium nanoparticles (SeNPs), a highly promising candidate as a nutrient fortificant and food additive, face challenges in stability and biosafety. These limitations hinder their application in the food industry. In this study, *Ganoderma lucidum* melanin (GLM) was utilized as a natural stabilizer. Three distinct types of GLM-stabilized SeNPs, termed GLM-SeNPs (S-GLM, D-GLM, and A-GLM), were subsequently synthesized via an ascorbic acid reduction method. The results showed that the prepared nanoparticles exhibited uniform particle size (55–75 nm) and good dispersibility. Among them, S-GLM possessed the highest selenium content (2598.90 mg/kg) and demonstrated the best stability. GLM-SeNPs significantly downregulated (*p* < 0.05) the mRNA expression of key pro-inflammatory cytokines (TNF-α, IL-6, and IL-1β) and upregulated (*p* < 0.05) the mRNA expression of the anti-inflammatory factor IL-10 in LPS-induced RAW264.7 macrophages. A potential mechanism underlying this effect may be the suppression of the NF-κB signaling pathway. In addition, GLM-SeNPs exhibited potent inhibitory effects against common foodborne pathogens. This study explores a potential novel strategy for the high-value utilization of natural functional components in food systems. These preliminary findings suggest GLM-SeNPs may have application potential in areas like functional beverages and food preservation. Further research is warranted to validate their feasibility in real food systems.

## 1. Introduction

Selenium is an essential trace element for the human body, existing in the form of selenoproteins [1]. It is widely involved in various physiological processes including immune regulation [2] and antioxidant defense [3], and is of great significance for the prevention and treatment of cardiovascular diseases [4], diabetes [5], and cancer [6]. Since the human body cannot synthesize selenium independently, it must be obtained through dietary sources [7], making a balanced diet crucial for maintaining adequate selenium levels. However, the extremely small gap between the effective dose and the toxic dose of selenium significantly limits its practical application [8,9]. Selenium intake should be maintained within the safe range of 60 μg (recommended) to 400 μg (upper limit) daily. Therefore, exceeding this range may lead to selenium toxicity and associated health risks [10]. Studies have shown that the toxicity of selenium is highly dependent on its chemical form [11], which has driven the development of new types of selenium that are low in toxicity and high in efficiency. Wang et al. found that selenium nanoparticles (SeNPs) were equally effective as selenomethionine in enhancing key antioxidant enzyme activities but exhibited significantly lower toxicity [12]. Additionally, the LD_50_ of SeNPs (113.0 mg Se/kg) was approximately 7 times that of sodium selenite (15.7 mg Se/kg), confirming its markedly lower acute toxicity [13]. This provides a promising strategy for solving the application problems of selenium. Nevertheless, the practical application of SeNPs faces considerable challenges. Firstly, chemically synthesized SeNPs often suffer from poor stability, tendency to aggregate, and inconsistent particle size distribution [14]. Secondly, the synthesis process may involve toxic chemical reagents, leading to compromised biocompatibility and potential toxicity risks [15]. To overcome these bottlenecks and fully harness the application potential of SeNPs, it is particularly urgent to explore an environmentally friendly green synthesis strategy for producing highly stable SeNPs.

To address the stability and biosafety challenges encountered by SeNPs during their synthesis and application, green synthesis employing bioactive templates has emerged as an effective strategy. This type of template not only enhances the stability of SeNPs but also endows them with new physiological functions. Currently, various biomaterials, including polysaccharides [16], proteins [17], and amino acids [18], have been extensively studied. However, both types of templates exhibit limitations in their mechanisms of action: polysaccharides primarily rely on the covalent backbone, while proteins depend mainly on their natural conformation, which is susceptible to environmental perturbation, thereby carrying a risk of denaturation and loss of function. It is worth noting that melanin, a natural biological macromolecule composed of indole or phenolic structural units [19], has rarely been reported as a template for the synthesis of SeNPs. The unique chemical structure of melanin confers multiple biological activities, including antioxidation [20], antibacterial [21], anti-tumor [22], immune regulation [23], radiation protection [24], and photothermal properties [25], demonstrating its potential applications in food and agriculture. More importantly, the active groups, such as phenolic hydroxyl and amino groups [26], carried by melanin molecules can not only exert a reducing effect through electron transfer but also achieve a template effect based on their molecular structure. In the preparation of SeNPs, this method not only directs the selenium source for efficient conversion into SeNPs, reducing by-product generation, but also inhibits nanoparticle agglomeration through adsorption encapsulation, thereby significantly enhancing the stability of the SeNPs. The key lies in the abundant functional groups (e.g., hydroxyl, amino, carbonyl, and carboxyl groups) and the internal hierarchical structures (such as π-π stacking and hydrophobic domains) possessed by melanin. These features enable the synergistic reduction in the surface free energy of selenium nanoparticles through multiple modes of interaction, including hydrophobic effects, hydrogen bonding, and electrostatic interactions [27]. Thereby, this provides a more robust stabilization strategy compared to traditional templates that rely primarily on a single type of interaction. Among various sources of melanin, mushroom-derived melanin is particularly notable. Its safety has been demonstrated through long-term use, making it suitable for applications in fields such as food and medicine. Compared to the constrained availability of plant and animal extracts and the potential toxicity risks associated with microbial fermentation, mushroom-based melanin achieves an optimal balance among safety, functionality, and economy, providing an ideal platform for the green synthesis and large-scale application of SeNPs.

*Ganoderma lucidum*, valued as a precious macrofungus, has been used as both food and medicine in China for thousands of years, with its value well-documented [28]. Modern studies confirm its richness in diverse bioactive compounds [29], demonstrating functionalities such as immune regulation [30], antioxidant [31], anti-inflammatory [32], anti-cancer [33], hepatoprotective [34], anti-diabetic [35], and neuroprotective activities [36]. Despite this, research has predominantly centered on its polysaccharides and triterpenoids, while the abundant melanin in its fruiting bodies remains largely neglected. During deep processing, its melanin is discarded with cell wall residues as a mere by-product, leading to significant resource waste and a lost opportunity to develop it as a natural functional component and green processing aid. More importantly, studies on synthesizing SeNPs using GLM as a template are scarce. It remains unexplored whether GLM as a biological template can solve the stability and biocompatibility issues of conventional SeNPs, and whether the synergistic bioactivities (e.g., anti-inflammatory and antioxidant) of GLM and SeNPs surpass those of the individual components. These constitute the current gaps in this research field.

Based on the above analysis, this study proposes a clear and testable working hypothesis: utilizing discarded GLM as a template enables the successful preparation of highly stable and low-toxicity *G. lucidum* melanin selenium nanoparticles (GLM-SeNPs). The resulting GLM-SeNPs are expected not only to inherit the inherent bioactivities of both GLM and SeNPs—such as antioxidant and antibacterial properties—but also to exhibit synergy-enhanced functionalities. Consequently, GLM-SeNPs hold promising potential for applications in functional foods or food preservation systems. To verify this hypothesis, this study successfully prepared and stabilized GLM-SeNPs. The GLM-SeNPs were comprehensively characterized by ultraviolet-visible (UV-Vis) spectroscopy, Fourier transform infrared spectroscopy (FT-IR), and scanning electron microscopy (SEM). Furthermore, the stability under varying storage times, pH levels, and ionic strength was evaluated, along with the anti-inflammatory, antioxidant, and antimicrobial properties. This work provides a novel approach for the high-value utilization of *G. lucidum*.

## 2. Materials and Methods

### 2.1. Materials

The fruiting bodies of *G. lucidum* were obtained from the Edible Mushroom Center, Shanxi Agricultural University (Jinzhong, China). Sodium selenite, selenium dioxide, selenious acid, ethyl acetate, sodium hydroxide, hydrochloric acid, trichloromethane, and L-ascorbic acid were purchased from Aladdin Biochemical Technology Co., Ltd. (Shanghai, China). Fetal bovine serum (FBS), phosphate-buffered saline (PBS), and dimethyl sulfoxide (DMSO) were purchased from Shanghai Yuanye Bio-Technology Co., Ltd. (Shanghai, China). Lipopolysaccharide (LPS; Solarbio Co., Ltd., Beijing, China; Batch No. 3550331029) was used in this study. High-glucose DMEM medium and all assay kits were also purchased from Solarbio Co., Ltd. (Beijing, China).

### 2.2. Extraction of G. lucidum Melanin (GLM)

Following the method of Hou et al. [37], GLM was extracted from *G. lucidum* fruiting bodies. Briefly, the powdered sample was dissolved in 1.25 mol/L NaOH. After centrifugation, the supernatant was acidified to pH 1.5 with HCl and incubated at 80 °C. The resulting precipitate was collected by centrifugation and washed with distilled water to neutrality. Subsequent purification steps were performed according to our established laboratory protocol [38]. The precipitate was then successively washed with chloroform and ethyl acetate, 95% ethanol, and 75% ethanol to remove impurities. Finally, the purified melanin was dissolved in 0.1 mol/L NaOH, neutralized to pH 7.0, and subjected to dialysis and lyophilization to obtain GLM. The crude melanin was purified via gel filtration (Sephadex G-15, 2.5 × 30 cm) eluted with 10 mM NaHCO_3_. The collected major fraction was dialyzed and lyophilized, yielding purified GLM.

### 2.3. Preparation of GLM-SeNPs

The GLM solution at a specific concentration was mixed with Na_2_SeO_3_, SeO_2_, and H_2_SeO_3_. The mixture was then magnetically stirred for 20 min. Subsequently, a Vc solution was added, and the reaction was conducted at 50 °C for 4 h until a stable orange-red solution formed. The mixture was then incubated for 48 h. After freeze-drying, GLM-SeNPs were obtained.

### 2.4. Determination of Selenium Content

Inductively coupled plasma optical emission spectrometry (ICP-OES) was used to quantify selenium in GLM-SeNPs. Precisely weighed samples in 150 mL tubes were digested on a digestion block with 9 mL 99.8% HNO_3_ and 1 mL 2% HClO_4_ (mixed acid solution). If the digest turned brownish-black, additional HNO_3_ was added dropwise until a colorless, transparent solution (complete digestion) was obtained. After cooling, the digest was transferred to a 10 mL volumetric flask, made up to volume with ultrapure water, and filtered through a 0.45 μm filter. Measurements were performed on an ICP-OES (Agilent 7800; Agilent Technologies, Santa Clara, CA, USA) in axial view mode at selenium’s characteristic wavelength (196.026 nm).

### 2.5. Characterization of GLM-SeNPs

A variety of analytical methods, referring to the approach of Subhash et al. [39], were employed for the characterization of S-GLM, D-GLM, A-GLM, and GLM. The particle size distribution and polydispersity index (PDI) were determined using dynamic light scattering (Malvern, Zetasizer ZS90, Malvern, UK). UV-Vis absorption spectra were recorded in the wavelength range of 200–800 nm using a spectrophotometer (Agilent, Cary 60, Santa Clara, CA, USA). FT-IR spectroscopy (Thermo Scientific, Nicolet iS5, Waltham, MA, USA) was conducted in the range of 500–4000 cm^−1^; the freeze-dried sample powder was thoroughly ground, mixed with potassium bromide at a mass ratio of 1:100, and pressed into a transparent sheet for measurement. Microscopic morphology was observed using SEM (JEOL, JSM-IT800, Singapore). Thermal stability was assessed via thermogravimetric analysis (Setaram, THEMYS, Lyon, France), with samples heated from 25 °C to 600 °C at a rate of 10 °C/min.

### 2.6. Effects of Different Influencing Factors on the Stability of GLM-SeNPs

To examine the effects of pH, ion concentration, and storage duration on the stability of GLM-SeNPs solutions were prepared with varying pH levels (2–10), NaCl (0.25–4 mg/mL) concentrations, and CaCl_2_ (0.25–4 mg/mL) concentrations, respectively. The sample solution was stored at 4 °C for 30 d, with particle size and PDI measurements taken at various time points during storage. All samples were thoroughly mixed prior to testing.

### 2.7. Effect of GLM and GLM-SeNPs on Survival Rate of RAW264.7 Cells

According to the method described by Wu et al. [40], the effects of GLM and GLM-SeNPs on the survival rate of RAW264.7 cells were evaluated using the MTT assay. RAW264.7 cells in the logarithmic growth phase were seeded into a 96-well plate at a density of 1 × 10^6^ cells/mL (100 µL/well) and incubated for 12 h. After removing the supernatant, 100 µL of fresh medium was added to the blank control group, and 100 µL of medium containing different concentrations of GLM or GLM-SeNPs was added to the experimental groups. Following 24 h of treatment, the supernatant was discarded, and 10 µL of MTT solution (5 mg/mL) was added to each well. After 4 h of incubation at 37 °C, the supernatant was removed, and 150 µL of DMSO was added to dissolve the formazan crystals. Absorbance was measured at 490 nm. Cell viability was calculated using the following formula:

(1) $$Cell\;survival\;rate\left(\%\right)=\frac{A_{0}}{A_{1}}\times 100$$

*A*_0_ represents the absorbance of the experimental group, while *A*_1_ denotes the absorbance of the blank group.

### 2.8. Effects of GLM and GLM-SeNPs on LPS-Induced Survival Rate of RAW264.7 Cells

Cells were cultured following previously established methods [41]. To investigate the protective effects of GLM and GLM-SENPs on LPS-induced inflammatory injury in RAW264.7 cells, the cell survival rate was assessed using the experimental method described in Section 2.7, both the model group and the treatment group were exposed to LPS-containing medium.

### 2.9. Effects of GLM and GLM-SeNPs on LPS-Induced Changes in NO Levels in RAW264.7 Cells

Slightly modified from the method of Li et al. [42], fresh medium was added to the blank group, LPS-containing medium was added to the model group, and medium containing different concentrations of GLM and GLM-SeNPs was added to the sample group. After 4 h, add LPS-containing medium to the sample group. After incubating for an additional 24 h, we collected the supernatant and measured the NO concentration.

### 2.10. Influence of Secretion Levels of Cellular Inflammatory Factors

Blank, model, and sample groups were established (cells were pretreated with 50, 100, 200, and 400 μg/mL of fresh medium for 4 h, followed by the addition of LPS medium), and the supernatant was collected after 24 h. The assay was performed by referring to the instructions in the kit.

### 2.11. Quantitative Reverse Transcription Polymerase Chain Reaction (qRT-PCR)

Following the method of Feng et al. [43], the expression level of the target gene was analyzed using qRT-PCR (BIO-RD, CFX96, Hercules, CA, USA). The relative expression levels of genes were calculated using the 2^−ΔΔCt^ method and normalized with GAPDH as the internal reference gene. Table 1 presents the primer sequences for each gene.

### 2.12. Determination of Antioxidant Activity

The antioxidant-related properties of GLM and GLM-SeNPs were determined using the method described by Feng et al. [44]. In all assays, ascorbic acid was used as the positive control, while the solvent used for sample preparation served as the negative control to rule out its interference. A blank containing only the reaction reagents was included for baseline calibration. The ABTS free radical scavenging rate and total reducing power were determined in strict accordance with the instructions provided in the corresponding reagent kits. The hydroxyl radical scavenging rate assay involves preparing each sample at concentrations of 0.25, 0.5, 1, 2, and 4 mg/mL, mixing them with the reagent in the specified proportions, and incubating the mixture at 37 °C for 30 min. Absorbance measurements are then taken at 510 nm for evaluation. In experimental group *A*_1_, DPPH solution was added; in control group *A*_2_, anhydrous ethanol solution was added; in blank group *A*_0_, distilled water and DPPH solution were added. To determine the DPPH scavenging activity, 1 mL of each reaction mixture was transferred to a cuvette after 15 min of reaction. The absorbance was then measured at 517 nm, using a mixture of distilled water and anhydrous ethanol for instrument zeroing. The DPPH scavenging activity was calculated as follows:

(2) $$Clearance\;rate\left(\%\right)=\left(1-\frac{A_{1}-A_{2}}{A_{0}}\right)\times 100$$

where *A*_0_, *A*_1_ and *A*_2_ were absorbance of blank group, experimental group and control group, respectively.

### 2.13. Determination of Bacteriostatic Activity

The bacterial suspensions of *Escherichia coli*, *Staphylococcus aureus*, and *Bacillus subtilis* were prepared using the Kirby-Bauer filter paper strip method for the determination of bacterial inhibition. Pipette 90 μL of bacterial suspension onto LB agar and spread it evenly across the plate using a sterile glass spreader. Place filter paper discs saturated with GLM and GLM-SeNPs onto the plate, using sterile water-saturated filter paper discs as the negative control. The plates were subsequently placed in a standard incubator, and the zones of inhibition were measured at 24 h intervals for 72 h. The plates were then maintained in the incubator. The zones of inhibition were measured at 24 h intervals up to 72 h. To determine the minimum inhibitory concentration (MIC) of GLM and GLM-SeNPs, both solutions were subjected to twofold serial dilution, and the aforementioned procedure was repeated.

### 2.14. Statistical Analysis

Data are presented as the mean ± standard deviation (SD) of at least three independent experiments. Prior to statistical comparison, the assumptions of normality and homogeneity of variances were verified using the Shapiro–Wilk test and Levene’s test, respectively. Statistical significance among groups was determined by one-way analysis of variance (ANOVA). When a significant main effect was observed (*p* < 0.05), Duncan’s new multiple range test was applied for post hoc pairwise comparisons. All statistical analyses were performed using IBM SPSS Statistics (Version 27.0; IBM Corp., Armonk, NY, USA). Significant differences are indicated by distinct lowercase superscript letters in the figures, which are based on the results of the Duncan’s test. Graphs were generated using OriginPro 2018 (OriginLab Corporation, Northampton, MA, USA).

## 3. Results and Discussion

### 3.1. Determination of the Size, PDI and Selenium Content of GLM-SeNPs

**Figure 1:**
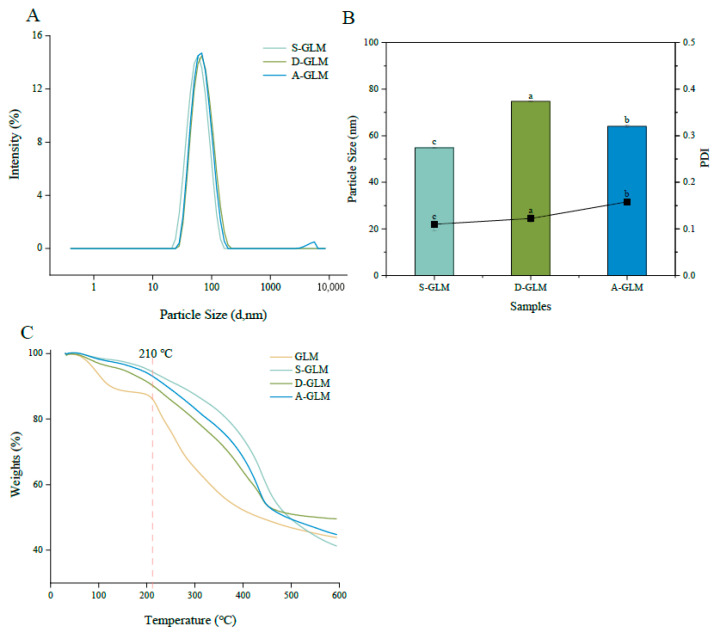
Physicochemical property analysis of GLM and GLM-SeNPs. (**A**) Particle size distribution of GLM-SeNPs. (**B**) Determination of particle size and polydispersity index of GLM-SeNPs. (**C**) Thermogravimetric analysis of GLM and GLM-SeNPs. Note: Values are mean ± SD. Different superscript letters within a column denote significant differences (*p* < 0.05) based on one-way ANOVA and Duncan’s multiple range test (*n* = 3). Nor-mality and homogeneity of variance assumptions were met.

Particle size and the PDI are critical parameters influencing the stability and dispersion of nanoparticles. On the one hand, the large size of nanoparticles leads to aggregation; on the other hand, it results in greater gravitational influence, accelerated sedimentation, and reduced stability. A higher PDI indicates less uniformity among the nanoparticles. As shown in Figure 1A,B, the particle sizes are 54.81 nm for S-GLM, 74.74 nm for D-GLM, and 64.02 nm for A-GLM, with S-GLM exhibiting the smallest particle size (*p* < 0.05). These sizes are notably smaller than the *Oudemansiella radicata* polysaccharide-based SeNPs (ORP1-SeNPs and ORP2-SeNPs) developed by Liu et al., which had average diameters of 106.28 nm and 117.04 nm, respectively [45]. A PDI below 0.3 indicates that the nanoparticle distribution is more uniform. The PDIs of S-GLM, D-GLM, and A-GLM are 0.11, 0.12, and 0.16, respectively, all of which are below 0.3, demonstrating that the distributions of GLM-SeNPs are highly uniform. In addition to these favorable physical characteristics, the selenium content was determined. As shown in Table 2, the levels were 2598.90 mg/kg for S-GLM, 1771.41 mg/kg for D-GLM, and 1105.78 mg/kg for A-GLM. These values demonstrate the successful incorporation of selenium into the nanoparticles.

### 3.2. Thermogravimetric and SEM Analysis

**Figure 2:**
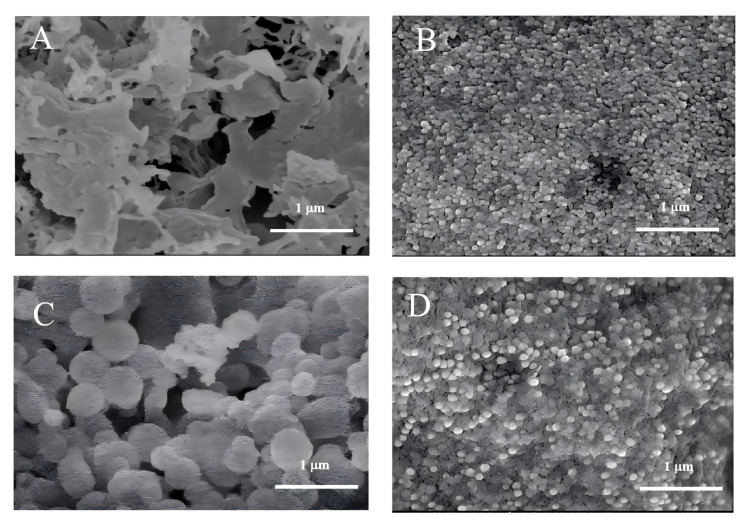
Scanning electron microscope images of GLM and GLM-SeNPs (×30,000, scale bar = 1 μm). (**A**) GLM, (**B**) S-GLM, (**C**) D-GLM, (**D**) A-GLM.

The thermal stability of the samples was evaluated by thermal analysis (Figure 1C), which showed that GLM underwent a two-step weight loss process. The first step (100–210 °C) corresponded to the evaporation of bound water [46], while the major weight loss in the second stage peaked at around 594 °C, with a total mass loss of 43.88%. This is attributed to the cleavage of covalent bonds between aromatic rings and the thermal decomposition of the polymer backbone in GLM [47]. The weight loss for S-GLM, D-GLM, and A-GLM was concentrated near 590 °C, with mass loss rates of 41.25%, 49.54%, and 44.77%, respectively. Similarly, Cao et al. reported that polysaccharide-functionalized SeNPs from *Grateloupia Livida* also exhibited a primary weight loss stage within a comparable high-temperature region [48]. This similarity supports the view that functionalized SeNPs stabilized by biomacromolecules generally possess excellent thermal stability. Furthermore, the decomposition temperature of GLM-SeNPs (≈590 °C) greatly exceeds the temperatures typical of common food processing (e.g., baking at 200 °C, sterilization at 121 °C). This substantial thermal margin indicates a strong potential for the application of GLM-SeNPs in heat-processed foods. Overall, the combination of SeNPs and GLM improved the overall thermal stability and showed certain heat resistance. As shown in Figure 2, SEM observations revealed that GLM exhibited a porous cavity structure. In contrast, the GLM-SeNPs formed spherical nanoparticles with a uniform distribution. SeNPs stabilized by *Ribes nigrum* L. polysaccharides have also been reported to exhibit a highly similar spherical morphology [49]. This confirms that natural biomacromolecules (e.g., polysaccharides or melanin-like substances) can generally act as both templates and stabilizers during the synthesis of SeNPs through their abundant surface functional groups, thereby tending to induce the formation of thermodynamically stable spherical structures. The uniform and stable nature of these SeNPs is attributed to the abundant functional groups and high specific surface area of the GLM molecular structure.

### 3.3. UV-Vis Spectroscopy and FT-IR Spectral Analysis

**Figure 3:**
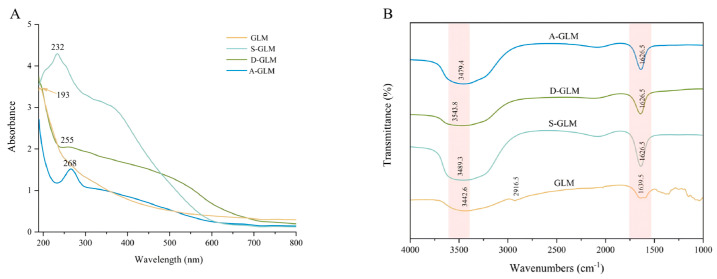
Chemical structure characterization of GLM and GLM-SeNPs. (**A**) UV-Vis absorption spectra and (**B**) FT-IR spectra of GLM and GLM-SeNPs.

As shown in Figure 3A, the maximum absorption wavelength of GLM is 193 nm, which is comparable to the characteristic absorption peaks of *Auricularia auricula-judae* melanin (210 nm) [50] and *Boletus aereus* melanin (214 nm) [51], confirming its typical melanin-like spectral properties. GLM exhibits strong absorption in the ultraviolet region (200–380 nm), followed by a gradual decrease in absorbance across the visible region (400–700 nm). The characteristic absorption peak of SeNPs is 263 nm [52], while those of S-GLM and D-GLM are 232 nm and 255 nm, respectively. These results indicate that the different preparation methods may lead to variations in the absorption peaks. The characteristic absorption peak of A-GLM was observed at 268 nm, which closely matches that of the *Morinda officinalis* polysaccharide SeNPs (Se-MOP, 266 nm) [53]. This similarity implies that the A-GLM preparation method may yield similar morphology and surface characteristics to Se-MOP. These results indicate that GLM-SeNPs were successfully prepared.

FT-IR is an essential technique for identifying functional groups and chemical bonds in compounds based on the positions and intensities of their characteristic absorption peaks [54]. As shown in Figure 3B, the absorption peaks of GLM closely resembled those of GLM-SeNPs, suggesting that no new covalent bonds had formed between GLM and SeNPs [55]. The absorption peak of GLM at 3442 cm^−1^ corresponded to the O-H stretching vibration [56], while the small peak at 2916 cm^−1^ was attributed to the stretching vibration of the aliphatic C-H group [57]. The most intense absorption peak at 1639 cm^−1^ was a typical characteristic absorption peak of fungal melanin. This phenomenon resulted from the stretching vibration of the C=O group, the C=C group, or the bending vibration of the N-H group [38]. When GLM bound to SeNPs, the D-GLM peak shifted from 3442 cm^−1^ to 3543 cm^−1^, and the carbonyl absorption peak weakened, indicating that the carboxyl groups in GLM and the conjugated system reacted with SeO_2_ [58]. The S-GLM and A-GLM shifted from 3442 cm^−1^ to 3489 cm^−1^ and 3479 cm^−1^, respectively, indicating that the OH group of GLM interacted with the Se atom in SeNPs through a hydrogen bond [59]. Sharp, narrow absorption peaks appeared at 1626 cm^−1^ for GLM-SeNPs, corresponding to the C=O stretching vibration in the amide I bond [60]. Collectively, the above FT-IR results are consistent with previous studies, confirming that GLM-SeNPs were successfully synthesized.

### 3.4. The Stability of GLM-SeNPs

As shown in Table 3, Table 4, Table 5 and Table 6, GLM-SeNPs exhibited significant differences in pH, ionic strength and storage stability. Within the pH range of 5–10, all samples remained stable with particle sizes below 72 nm and PDI values lower than 0.3, among which S-GLM showed the smallest particle size. However, under strongly acidic conditions, the particle size increased significantly to approximately 2000 nm with a PDI above 0.6, indicating severe aggregation in the system, which may be attributed to changes in the surface electrochemical properties of SeNPs and the potential formation of new chemical bonds. This aggregation behavior clearly limits GLM-SeNPs’ direct application in highly acidic food matrices, such as citrus-based beverages, fermented foods, or dressings with low pH. Under Na^+^ concentrations ranging from 0.25 to 4 mg/mL, the particle size of S-GLM remained stable at around 50 nm with the lowest PDI. This indicates its excellent salt tolerance and potential for application in salted products. In contrast, the particle sizes of D-GLM and A-GLM initially increased and then decreased with rising Na^+^ concentration, which may be due to the adsorption of NaCl on the particle surfaces, leading to the formation of transient large particles [61]. When the Ca^2+^ concentration reached 4 mg/mL, all samples exhibited severe aggregation, with particle sizes increasing to several thousand nanometers and PDI exceeding 0.4, primarily due to the chelation between Ca^2+^ and GLM, which disrupted their interaction with SeNPs. In terms of storage stability, S-GLM consistently maintained the smallest particle size and the lowest PDI throughout the storage period, demonstrating optimal long-term stability. The stability variations observed among the GLM-SeNPs primarily stem from the distinct chemical nature of their selenium precursors, which establish fundamentally different reaction microenvironments. These conditions subsequently alter the exposure of active sites on the GLM backbone, leading to differences in its final structural conformation and interfacial behavior—including how it binds to and coats the SeNPs. These distinct interfacial characteristics ultimately account for the significant divergence in stability among S-GLM, D-GLM, and A-GLM. In summary, S-GLM exhibited exceptional stability under the controlled conditions tested in this study. However, its behavior in real, complex food matrices—which contain proteins, polysaccharides, lipids, and variable pH—remains unverified. Therefore, direct extrapolation of these stability results to practical food applications is not currently justified. The stability demonstrated here under ideal laboratory conditions establishes a necessary foundation and strongly motivates systematic future research to evaluate its performance in targeted food systems.

### 3.5. Anti-Inflammatory Activity of GLM and GLM-SeNPs in LPS-Induced RAW 264.7 Cells

#### 3.5.1. Effects of GLM and GLM-SeNPs on RAW264.7 Cells Viability

**Figure 4:**
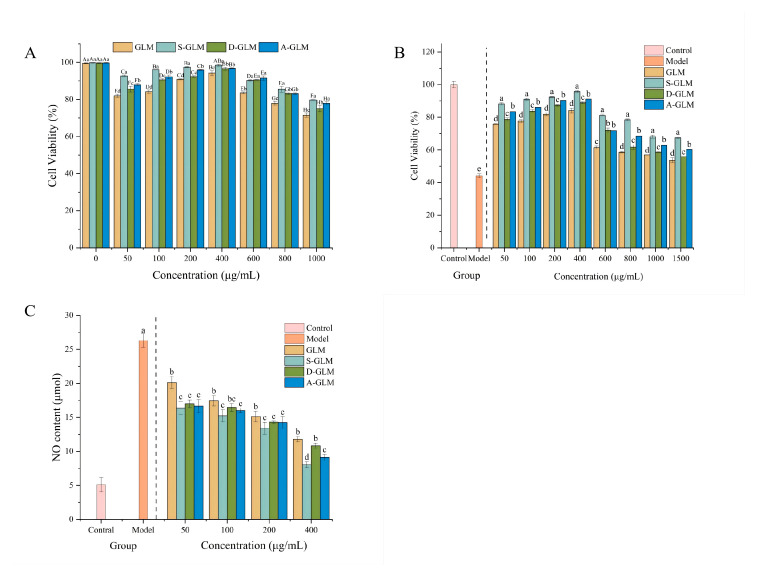
(**A**) The effects of GLM and GLM-SeNPs on the cell viability of RAW264.7 cells under normal and (**B**) LPS-induced conditions. (**C**) The effects of GLM and GLM-SeNPs on NO Production in LPS-Induced RAW264.7 Cells. Data are presented as mean ± SD (*n* = 3). Significant differences (*p* < 0.05) among groups were determined by one-way ANOVA followed by Duncan’s multiple range test, and are indicated by different lowercase letters, with different uppercase letters representing inter-group significance at the same level.

The biocompatibility and protective effects of GLM and GLM-SeNPs were systematically evaluated using the MTT assay. Initial assessment under normal culture conditions revealed low cytotoxicity for all samples (Figure 4A). The cell survival rate exceeded 94.18% at 400 μg/mL and remained above 83.53% even at 600 μg/mL, demonstrating excellent biocompatibility. The potential of the samples to protect against LPS-induced inflammatory injury was further investigated. As shown in Figure 4B, LPS significantly decreased cell viability. However, pretreatment with GLM-SeNPs (50–400 μg/mL) effectively counteracted this LPS-induced cytotoxicity, resulting in survival rates all above 75.82%. Consequently, the concentration range from 50 to 400 μg/mL was selected for subsequent mechanistic studies.

#### 3.5.2. Effect of GLM and GLM-SeNPs on NO Production

Nitric oxide (NO) is a biologically active gaseous molecule closely associated with inflammatory responses. During acute inflammation, excessive NO exerts cytotoxic effects that can induce cellular damage and exacerbate the inflammatory process, which is linked to various health concerns. As shown in Figure 4C, LPS stimulation significantly increased NO production in RAW264.7 cells by 26.3% compared to the control group (*p* < 0.05), confirming successful inflammatory model establishment. Treatment with GLM and GLM-SeNPs significantly attenuated this LPS-induced NO overproduction in a concentration-dependent manner. At the concentration of 400 μg/mL, NO levels were markedly reduced to 11.79 μmol (GLM), 8.04 μmol (S-GLM), 10.82 μmol (D-GLM), and 9.12 μmol (A-GLM), respectively. This concentration-dependent NO suppression aligns with reports on various natural plant extracts, such as kiwifruit extract [62]. Building on this, the innovation of the present study lies in developing GLM-SeNPs as a novel composite system. The advantage of this system stems from the synergy between selenium and melanin, which not only leads to effective NO-inhibitory activity but, more importantly, demonstrates that constructing a nanocomposite can achieve a synergistic enhancement of anti-inflammatory activity between selenium and natural bioactive components. This finding provides a new strategy for developing potent anti-inflammatory functional food ingredients.

Notably, further correlation analysis revealed a very strong positive correlation between the level of NO production and particle size (r = 0.996), indicating that smaller particle size corresponds to more significant NO inhibition. However, this association did not attain formal statistical significance (*p* = 0.054). These results indicate that GLM and GLM-SeNPs exhibit significant anti-inflammatory activity by effectively inhibiting NO overproduction in activated macrophages, thereby protecting RAW264.7 cells from inflammatory damage. Although the reduction in NO serves as an important phenotypic indicator, this terminal event is insufficient to elucidate the upstream regulatory mechanisms. To explore these underlying mechanisms, we speculate that the inhibition of NO generation by GLM-SeNPs—particularly the highly stable S-GLM—may be attributed to their intervention in upstream inflammatory signaling, such as TLR4 activation, NF-κB pathway induction, or reactive oxygen species (ROS) accumulation. This proposed mechanism requires further experimental validation.

#### 3.5.3. Effect of GLM and GLM-SeNPs on Inflammatory Factor Levels

**Figure 5:**
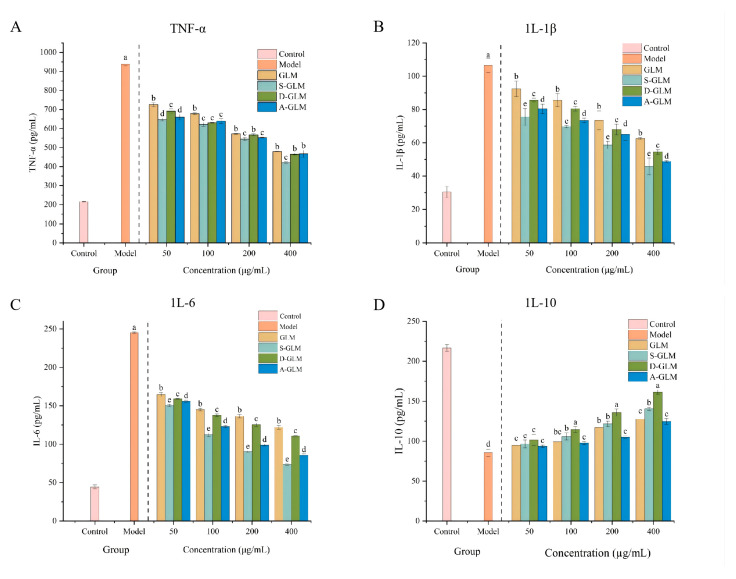
Effects of GLM and GLM-SeNPs on Inflammatory Factors in LPS-induced RAW264.7 cells. (**A**) TNF-α, (**B**) IL-1β, (**C**) IL-6, (**D**) IL-10. Data are presented as mean ± SD (*n* = 3). Significant differences (*p* < 0.05) among groups were determined by one-way ANOVA followed by Duncan’s multiple range test, and are indicated by different lowercase letters.

The in vitro anti-inflammatory activities of GLM and GLM-SeNPs were evaluated by measuring the levels of inflammatory factors in RAW264.7 cells. Tumor necrosis factor-alpha (TNF-α), a cytokine produced by various immune and non-immune cells, induces the release of IL-6 and IL-1β, representing one of the most potent inflammatory mediators [63]. As shown in Figure 5A–D, the LPS-induced model group exhibited significantly elevated levels of TNF-α, IL-6, and IL-1β compared to the blank group. Treatment with GLM and GLM-SeNPs reduced the levels of these pro-inflammatory factors to varying degrees. At a concentration of 400 μg/mL, the S-GLM treatment group demonstrated the most potent inhibitory effect, leading to a marked suppression in the release of TNF-α, IL-6, and IL-1β. This suppression of pro-inflammatory cytokine secretion at the protein level is consistent with the downregulation of their corresponding mRNA expression, as detailed in the following section.

The anti-inflammatory cytokine IL-10 plays a crucial role in suppressing inflammatory responses, making it beneficial for alleviating certain inflammatory and autoimmune conditions. The IL-10 level was 216.67 pg/mL in the blank group and decreased to 85.50 pg/mL in the model group. At 400 μg/mL, the D-GLM treatment group showed the highest IL-10 level (*p* < 0.05), indicating its unique capacity to enhance IL-10 secretion and thereby mitigate the inflammatory response. These protein-level data are in full agreement with the subsequent gene expression results, which show a notable upregulation of IL-10 mRNA, thereby reinforcing the finding at both functional and transcriptional levels. This finding supports the widely adopted approach of using bioactive macromolecules to enhance the bioactivity of SeNPs. For instance, Wang et al. [55] reported that *Poria cocos* polysaccharide-modified SeNPs (PCP-SeNPs) showed significantly stronger anti-inflammatory activity than the polysaccharide alone. Similarly, GLM-SeNPs demonstrated superior anti-inflammatory potential compared to GLM itself. The development of GLM-SeNPs not only confirms the effectiveness of using bioactive macromolecules to stabilize SeNPs but also expands the selection of functional ingredients available for the development of novel functional foods by introducing melanin as a promising new stabilizer, embodied in GLM-SeNPs.

#### 3.5.4. Effects of GLM and GLM-SeNPs on the mRNA Expression of Key Inflammatory Mediators

**Figure 6:**
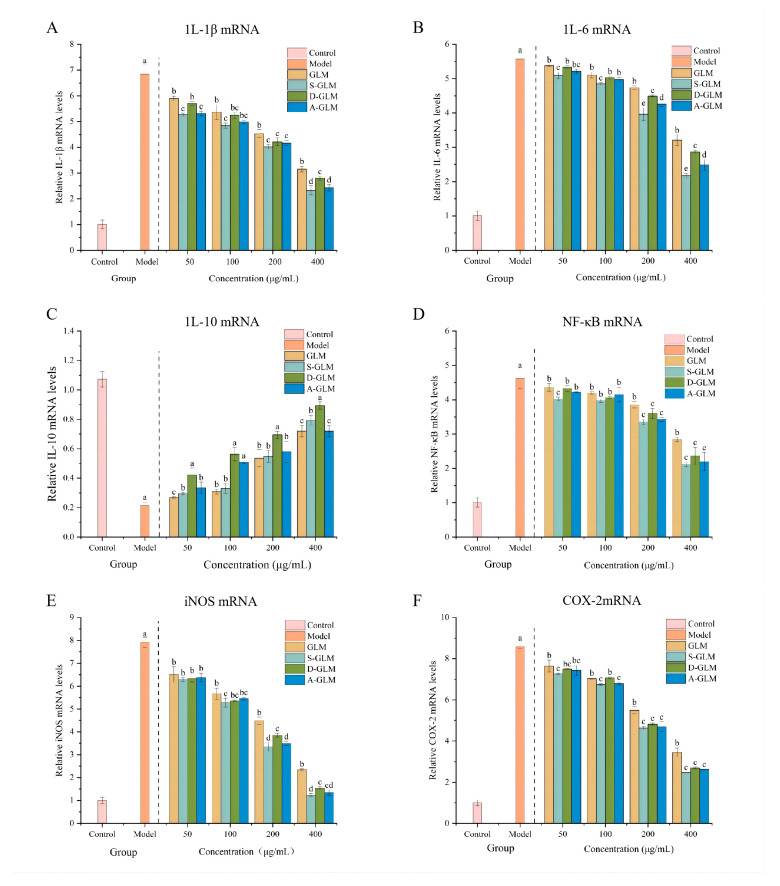
Effects of GLM and GLM-SeNPs on the mRNA expression of key inflammatory mediators in LPS-induced RAW264.7 cells. (**A**) IL-1β, (**B**) IL-6, (**C**) IL-10, (**D**) NF-κB, (**E**) iNOS, (**F**) COX-2. Data are presented as mean ± SD (*n* = 3). Significant differences (*p* < 0.05) among groups were determined by one-way ANOVA followed by Duncan’s multiple range test, and are indicated by different lowercase letters.

Consistent with the observed changes in cytokine protein levels, analysis at the mRNA level revealed that GLM and GLM-SeNPs significantly suppressed the expression of IL-1β and IL-6, while increasing IL-10 expression compared to the LPS model group (Figure 6A–C). Among them, S-GLM and A-GLM exhibited the strongest inhibition on IL-1β, whereas D-GLM showed the most pronounced promotion of IL-10 at 400 µg/mL. Notably, the IL-10 upregulation effect of GLM-SeNPs aligns with the anti-inflammatory trend reported for SeNPs stabilized by *G. lucidum* polysaccharides, confirming that biomolecules from this fungus (such as polysaccharides and melanin) are effective templates for bioactive SeNPs [64]. The anti-inflammatory activity was further linked to the modulation of the NF-κB signaling pathway. Specifically, both GLM and GLM-SeNPs reversed the LPS-induced upregulation of NF-κB (Figure 6D), leading to a corresponding decrease in the expression of its downstream effectors, iNOS and COX-2 (Figure 6E,F). This mechanistic insight strengthens the evidence for the potent anti-inflammatory functionality of GLM-SeNPs at the cellular level. Notably, GLM-SeNPs generally exhibited stronger regulatory effects than GLM alone. Together, the confirmed anti-inflammatory activity and the elucidated NF-κB-related mechanism provide a scientific basis for considering GLM-SeNPs as promising functional ingredients.

It should be noted that the anti-inflammatory activity observed in this study was evaluated using in vitro cell models, which cannot fully simulate key in vivo physiological processes such as gastrointestinal digestion, systemic absorption, and immune modulation via the gut microbiota axis. Consequently, the bioavailability and long-term dietary safety of GLM-SeNPs have not been established. In subsequent studies, it will be necessary to verify their in vivo anti-inflammatory efficacy and molecular targets using murine inflammation models, and to conduct food-relevant toxicological assessments in animals. Such work will help clarify safe dosage ranges and metabolic pathways, thereby providing critical support for the potential application of GLM-SeNPs as functional food ingredients.

### 3.6. Evaluation of In Vitro Antioxidant Activity

**Figure 7:**
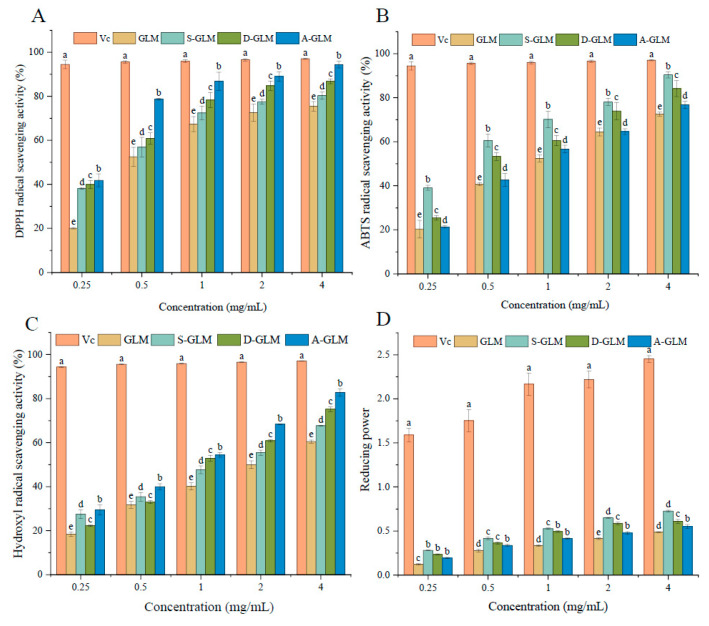
Antioxidant activity of GLM and GLM-SeNPs against the following: (**A**) DPPH radical, (**B**) ABTS radical, (**C**) hydroxyl radical, (**D**) reducing power. Data are presented as mean ± SD (*n* = 3). Significant differences (*p* < 0.05) among groups were determined by one-way ANOVA followed by Duncan’s multiple range test, and are indicated by different lowercase letters.

Both GLM and GLM-SeNPs exhibited a concentration-dependent increase in activity across all antioxidant assays. In the DPPH assay (Figure 7A), the scavenging capacity at 4 mg/mL followed the order: A-GLM (94.34%) > D-GLM (86.80%) > S-GLM (80.31%) > GLM (75.44%), indicating that selenization significantly enhanced the antioxidant capacity of the biopolymer [65]. In the ABTS assay (Figure 7B), S-GLM showed the highest scavenging rate (90.36%) at the same concentration, which may be attributed to its smaller nanoparticle size and larger specific surface area, favoring the exposure of active sites [66]. The hydroxyl radical scavenging assay (Figure 7C) revealed that A-GLM had the highest efficiency (82.78%), significantly surpassing D-GLM (75.18%), S-GLM (67.69%), and GLM (60.48%). The total reducing power assay (Figure 7D) further demonstrated that A-GLM possessed the strongest electron-donating ability (absorbance 0.73). Notably, correlation analysis indicated that the antioxidant indices were only weakly correlated with particle size (|r| = 0.418–0.629), and lacked statistical significance (*p* > 0.05). This suggests that the antioxidant efficacy of GLM-SeNPs is not primarily governed by their nanoscale dimensions but is more likely rooted in the inherent redox-active functional groups (e.g., phenolic hydroxyl and quinone groups) of GLM. These groups can act as electron donors to directly quench free radicals via electron transfer, thereby providing stable antioxidant capacity [67]. Therefore, the superior antioxidant performance of GLM-SeNPs can be principally attributed to the intrinsic chemical properties of the GLM carrier. This finding provides a clear rationale for further investigation into their potential as antioxidant ingredients for functional foods.

### 3.7. Effects of GLM and GLM-SeNPs on Antibacterial Activity

The antibacterial efficacy of GLM and GLM-SeNPs was quantitatively assessed. The MIC values, presented in Table 7, confirmed the superior potency of the GLM-SeNPs. As shown in Table 8, at a concentration of 50 mg/mL, both GLM and GLM-SeNPs inhibited the growth of *Escherichia coli*, *Staphylococcus aureus*, and *Bacillus subtilis*. GLM produced an inhibition zone of only 15.60 ± 0.59 mm, whereas S-GLM, D-GLM, and A-GLM exhibited markedly stronger activity, with inhibition zones of 19.30 ± 0.69 mm, 21.03 ± 0.66 mm, and 23.68 ± 0.35 mm, respectively. It has been reported that the antibacterial efficacy of nanoparticles is size-dependent; smaller sizes provide a larger contact area, facilitating penetration of the bacterial membrane, inducing massive ROS production, disrupting cellular functions, and ultimately leading to bacterial death [68]. However, correlation analysis revealed that in the GLM-SeNPs investigated in this study, the antibacterial activity showed only weak to moderate correlation with particle size (r = 0.142–0.587). This suggests that the connection between structure and activity may be more complex and not solely governed by the nanoscale dimensions of the composite particles. Therefore, we speculate that the outstanding antibacterial performance of GLM-SeNPs—particularly the pronounced effect against Gram-positive bacteria—primarily stems from the synergistic interaction between GLM and SeNPs. This synergy may be manifested as follows: GLM initially disrupts the bacterial cell membrane through hydrophobic interactions and electrostatic forces, thereby facilitating the intracellular internalization of SeNPs [19]. Subsequently, the internalized SeNPs trigger a burst of ROS. This process may synergize with the oxidative stress mediated by the inherent redox-active functional groups of GLM, leading to cumulative oxidative damage that ultimately compromises key intracellular targets such as proteins and DNA [69]. Notably, the efficiency of this synergistic antibacterial effect is strongly modulated by the bacterial cell wall structure: Gram-positive bacteria lack an outer membrane barrier, and their surface properties favor the initial action of GLM-SeNPs, thereby enhancing the synergistic bactericidal efficiency. This directly explains why their inhibitory effect against Gram-positive bacteria such as *S. aureus* is generally stronger than that against Gram-negative bacteria such as *E. coli*, which possess an outer membrane barrier.

## 4. Conclusions

In summary, this study successfully developed a novel strategy for preparing SeNPs using GLM as a stabilizer. Systematic characterization and bioactivity evaluation demonstrated that the GLM-SeNPs exhibit significant antioxidant, anti-inflammatory, and antimicrobial activities in in vitro models. The anti-inflammatory effect of GLM-SeNPs may be associated with the modulation of signaling pathways such as NF-κB. These findings confirm the feasibility of fungal melanin as a functional stabilizer for bioactive food ingredients, thereby providing a new perspective for novel selenium-based functional ingredients. Building upon these foundational in vitro findings, our future research will systematically evaluate the practical applicability of GLM-SeNPs. This will include assessing their compatibility and processing stability within real food systems, as well as investigating key parameters for human health benefits, such as bioavailability after digestion and in vivo efficacy. These studies are essential to strengthen the scientific foundation for the practical application of GLM-SeNPs as promising functional food ingredients.

## Figures and Tables

**Table 1:** Primer sequence.

Primer Name	Froward Primer (5′-3′)	Reverse Primer (5′-3′)
GAPDH	CACTCACGGCAAATTCAACGGCACA	GACTCCACGACATACTCAGCAC
IL-Iβ	GTGGCAGCTACCTGTGTGTCTT	GGGAGCCTGTAGTGCAGTTGT
IL-6	CTGCAAGAGACTTCCATCCAG	AGTGGTATAGACAGGTCTGTTGG
IL-10	CTTACTGACTGGCATGAGGATCA	GCAGCTCTAGGAGCATGTGG
NF-κB	ATGGCAGACGATGATCCCTAC	CGGAATCGAAATCCCCTCTGTT
iNOS	GTTCTCAGCCCAACAATACAAGA	GTGGACGGGTCGATGTCAC
COX-2	TGCACTATGGTTACAAAAGCTGG	TCAGGAAGCTCCTTATTTCCCTT

**Table 2:** Determination of selenium content in GLM-SeNPs.

Sample	Selenium Content (mg/kg)	Solution State
S-GLM	2598.90	colorless
D-GLM	1771.41	colorless
A-GLM	1105.78	colorless

**Table 3:** Particle Size and PDI of GLM and GLM-SeNPs as a Function of pH.

	S-GLM		D-GLM		A-GLM	
pH	Particle Size (nm)	PDI	Particle Size (nm)	PDI	Particle Size (nm)	PDI
2	2219.67 ± 0.8 ^a^	0.64 ± 0.01 ^c^	1470.67 ± 1.76 ^c^	0.74 ± 0.01 ^a^	1606.67 ± 1.45 ^b^	0.68 ± 0.01 ^b^
3	1062.67 ± 1.2 ^a^	0.65 ± 0.02 ^a^	80.71 ± 0.18 ^b^	0.67 ± 0.01 ^a^	70.22 ± 0.83 ^c^	0.47 ± 0.01 ^b^
4	57.22 ± 0.39 ^c^	0.16 ± 0.01 ^c^	126.17 ± 0.89 ^a^	0.40 ± 0.01 ^a^	66.87 ± 0.56 ^b^	0.36 ± 0.01 ^b^
5	54.37 ± 0.08 ^c^	0.11 ± 0.01 ^b^	71.59 ± 0.23 ^a^	0.17 ± 0.01 ^a^	65.54 ± 0.43 ^b^	0.16 ± 0.01 ^a^
6	54.63 ± 0.09 ^c^	0.11 ± 0.01 ^c^	65.65 ± 0.32 ^a^	0.17 ± 0.01 ^a^	64.94 ± 0.12 ^b^	0.14 ± 0.01 ^b^
7	54.72 ± 0.19 ^c^	0.11 ± 0.01 ^a^	65.96 ± 0.16 ^a^	0.13 ± 0.01 ^a^	65.19 ± 0.11 ^b^	0.15 ± 0.01 ^a^
8	55.62 ± 0.05 ^c^	0.12 ± 0.01 ^b^	66.94 ± 0.37 ^a^	0.14 ± 0.01 ^ab^	64.04 ± 0.25 ^b^	0.15 ± 0.01 ^a^
9	53.81 ± 0.16 ^c^	0.12 ± 0.01 ^a^	65.44 ± 0.17 ^a^	0.12 ± 0.01 ^a^	62.75 ± 0.11 ^b^	0.13 ± 0.01 ^a^
10	53.91 ± 0.03 ^c^	0.10 ± 0.01 ^a^	62.81 ± 0.39 ^a^	0.12 ± 0.01 ^a^	60.27 ± 0.31 ^b^	0.10 ± 0.01 ^a^

Note: Values are mean ± SD. Different superscript letters within a column denote significant differences (*p* < 0.05) based on one-way ANOVA and Duncan’s multiple range test (*n* = 3). Normality and homogeneity of variance assumptions were met.

**Table 4:** Particle Size and PDI of GLM and GLM-SeNPs as a Function of Na^+^ Concentration.

	S-GLM		D-GLM		A-GLM	
Concentration (mg/mL)	Particle Size (nm)	PDI	Particle Size (nm)	PDI	Particle Size (nm)	PDI
0.25	52.95 ± 0.24 ^c^	0.11 ± 0.01 ^c^	320.03 ± 2.11 ^a^	0.52 ± 0.01 ^a^	225.66 ± 2.08 ^b^	0.39 ± 0.03 ^b^
0.5	53.33 ± 0.38 ^c^	0.09 ± 0.02 ^c^	353.96 ± 2.02 ^a^	0.61 ± 0.01 ^a^	231.63 ± 0.47 ^b^	0.35 ± 0.02 ^b^
1	52.65 ± 0.45 ^c^	0.11 ± 0.01 ^c^	410.16 ± 1.31 ^a^	0.65 ± 0.03 ^a^	252.41 ± 0.89 ^b^	0.42 ± 0.04 ^b^
2	52.75 ± 0.54 ^c^	0.11 ± 0.01 ^c^	351.56 ± 1.48 ^a^	0.63 ± 0.01 ^a^	275.61 ± 2.09 ^b^	0.46 ± 0.04 ^b^
4	53.57 ± 0.13 ^c^	0.11 ± 0.01 ^b^	395.43 ± 0.75 ^a^	0.45 ± 0.02 ^a^	248.13 ± 1.76 ^b^	0.45 ± 0.02 ^a^

Note: Values are mean ± SD. Different superscript letters within a column denote significant differences (*p* < 0.05) based on one-way ANOVA and Duncan’s multiple range test (*n* = 3). Normality and homogeneity of variance assumptions were met.

**Table 5:** Particle Size and PDI of GLM and GLM-SeNPs as a Function of Ca^2+^ Concentration.

	S-GLM		D-GLM		A-GLM	
Concentration (mg/mL)	Particle Size (nm)	PDI	Particle Size (nm)	PDI	Particle Size (nm)	PDI
0.25	217.91 ± 1.11 ^c^	0.19 ± 0.02 ^c^	3677.33 ± 1.66 ^a^	0.45 ± 0.04 ^b^	904.66 ± 1.44 ^b^	0.83 ± 0.03 ^a^
0.5	1129.33 ± 3.52 ^c^	0.46 ± 0.05 ^a^	2794.66 ± 4.05 ^b^	0.35 ± 0.02 ^b^	3574.66 ± 1.76 ^a^	0.47 ± 0.01 ^a^
1	1725.66 ± 3.38 ^b^	0.35 ± 0.04 ^b^	2432.66 ± 1.76 ^a^	0.26 ± 0.02 ^c^	2432.66 ± 1.76 ^a^	0.61 ± 0.06 ^a^
2	2009.66 ± 2.84 ^b^	0.31 ± 0.04 ^c^	1644.33 ± 2.40 ^c^	0.61 ± 0.01 ^b^	3076.13 ± 3.21 ^a^	0.71 ± 0.07 ^a^
4	4666.01 ± 3.21 ^a^	0.71 ± 0.04 ^a^	1418.33 ± 6.48 ^c^	0.57 ± 0.03 ^b^	2268.25 ± 4.16 ^b^	0.31 ± 0.05 ^c^

Note: Values are mean ± SD. Different superscript letters within a column denote significant differences (*p* < 0.05) based on one-way ANOVA and Duncan’s multiple range test (*n* = 3). Normality and homogeneity of variance assumptions were met.

**Table 6:** Particle Size and PDI of GLM and GLM-SeNPs as a Function of Storage Time.

	S-GLM		D-GLM		A-GLM	
Storage Days (d)	Particle Size (nm)	PDI	Particle Size (nm)	PDI	Particle Size (nm)	PDI
0	54.81 ± 0.27 ^c^	0.11 ± 0.02 ^c^	74.74 ± 0.09 ^a^	0.12 ± 0.01 ^b^	64.02 ± 0.52 ^b^	0.16 ± 0.01 ^a^
5	55.42 ± 0.82 ^c^	0.14 ± 0.01 ^b^	71.08 ± 0.44 ^a^	0.16 ± 0.01 ^a^	65.41 ± 0.41 ^b^	0.15 ± 0.01 ^a^
10	56.81 ± 0.52 ^c^	0.14 ± 0.01 ^c^	70.75 ± 0.55 ^a^	0.18 ± 0.01 ^a^	67.16 ± 0.76 ^b^	0.16 ± 0.01 ^b^
15	56.21 ± 0.64 ^c^	0.13 ± 0.01 ^b^	72.40 ± 0.85 ^a^	0.17 ± 0.01 ^a^	67.49 ± 0.73 ^b^	0.17 ± 0.01 ^a^
20	58.07 ± 0.65 ^c^	0.14 ± 0.01 ^b^	71.82 ± 0.54 ^a^	0.18 ± 0.01 ^a^	67.11 ± 1.58 ^b^	0.17 ± 0.01 ^a^
25	57.87 ± 0.45 ^c^	0.16 ± 0.01 ^c^	73.74 ± 0.68 ^a^	0.19 ± 0.01 ^a^	68.30 ± 0.72 ^b^	0.18 ± 0.01 ^b^
30	58.28 ± 0.42 ^c^	0.16 ± 0.01 ^b^	73.76 ± 0.62 ^a^	0.18 ± 0.01 ^a^	69.62 ± 0.47 ^b^	0.18 ± 0.01 ^a^

Note: Values are mean ± SD. Different superscript letters within a column denote significant differences (*p* < 0.05) based on one-way ANOVA and Duncan’s multiple range test (*n* = 3). Normality and homogeneity of variance assumptions were met.

**Table 7:** MIC of GLM and GLM-SeNPs against three bacterial species.

Bacteria	Concentration (mg/mL)	Diameter of the Inhibition Zone/mm			
		GLM	S-GLM	D-GLM	A-GLM
*Escherichia coli*	25	8.29 ± 0.03 ^d^	10.32 ± 0.19 ^c^	12.59 ± 0.33 ^b^	13.77 ± 0.19 ^a^
	12.5	6.49 ± 0.29 ^c^	8.14 ± 0.12 ^b^	8.37 ± 0.40 ^b^	9.31 ± 0.33 ^a^
	6.25	-	-	-	-
	3.13	-	-	-	-
*Staphylococcus aureus*	25	12.35 ± 0.4 ^d^	16.47 ± 0.31 ^c^	18.23 ± 0.27 ^b^	20.5 ± 0.31 ^a^
	12.5	8.89 ± 0.53 ^d^	10.51 ± 0.26 ^c^	14.89 ± 0.17 ^b^	17.16 ± 0.13 ^a^
	6.25	5.41 ± 0.48 ^c^	7.31 ± 0.15 ^b^	8.14 ± 0.11 ^a^	8.51 ± 0.42 ^a^
	3.13	4.83 ± 0.32 ^c^	5.12 ± 0.11 ^b^	5.52 ± 0.36 ^b^	6.38 ± 0.23 ^a^
*Bacillus subtilis*	25	10.41 ± 0.16 ^d^	12 37 ± 0.28 ^c^	14.41 ± 0.32 ^b^	16.22 ± 0.15 ^a^
	12.5	8.15 ± 0.11 ^c^	11.42 ± 0.23 ^b^	12.20 ± 0.19 ^a^	12.54 ± 0.27 ^a^
	6.25	5.33 ± 0.26 ^c^	8.51 ± 0.21 ^b^	9.09 ± 0.07 ^a^	9.44 ± 0.34 ^a^
	3.13	-	-	-	-

Note: Data are presented as mean ± SD (*n* = 3). Significant differences (*p* < 0.05) among groups were determined by one-way ANOVA followed by Duncan’s multiple range test, and are indicated by different lowercase letters.

**Table 8:** The effect of GLM, S-GLM, D-GLM and A-GLM on bacteriostatic activity.

Samples	Diameter of the Inhibition Zone/mm		
	*Escherichia coli*	*Staphylococcus aureus*	*Bacillus subtilis*
GLM	10.20 ± 0.78 ^c^	15.60 ± 0.59 ^d^	12.93 ± 0.02 ^c^
S-GLM	12.53 ± 0.32 ^b^	19.30 ± 0.69 ^c^	16.33 ± 0.72 ^b^
D-GLM	14.29 ± 0.88 ^a^	21.03 ± 0.66 ^b^	17.35 ± 0.80 ^b^
A-GLM	15.33 ± 0.66 ^a^	23.68 ± 0.35 ^a^	21.52 ± 0.58 ^a^

Note: Data are presented as mean ± SD (*n* = 3). Significant differences (*p* < 0.05) among groups were determined by one-way ANOVA followed by Duncan’s multiple range test, and are indicated by different lowercase letters.

## Data Availability

The original contributions presented in the study are included in the article; further inquiries can be directed to the corresponding authors.
